# Marine biogenics in sea spray aerosols interact with the mTOR signaling pathway

**DOI:** 10.1038/s41598-018-36866-3

**Published:** 2019-01-24

**Authors:** Jana Asselman, Emmanuel Van Acker, Maarten De Rijcke, Laurentijn Tilleman, Filip Van Nieuwerburgh, Jan Mees, Karel A. C. De Schamphelaere, Colin R. Janssen

**Affiliations:** 1 Laboratory of Environmental Toxicology and Aquatic Ecology, Environmental Toxicology Unit - GhEnToxLab, Ghent University, Campus Coupure, Coupure Links 653, Building F – 2nd Floor, Ghent, Belgium; 2 Flanders Marine Institute (VLIZ), InnovOcean site, Wandelaarkaai 7, 8400 Ostend, Belgium; 3 Laboratory for Pharmaceutical Biotechnology, Faculty of Pharmaceutical Sciences, Ghent University, Campus UZ, Ottergemse Steenweg 460, 9000 Ghent, Belgium

## Abstract

Sea spray aerosols (SSAs) have profound effects on our climate and ecosystems. They also contain microbiota and biogenic molecules which could affect human health. Yet the exposure and effects of SSAs on human health remain poorly studied. Here, we exposed human lung cancer cells to extracts of a natural sea spray aerosol collected at the seashore in Belgium, a laboratory-generated SSA, the marine algal toxin homoyessotoxin and a chemical inhibitor of the mammalian target of rapamycin (mTOR) pathway. We observed significant increased expression of genes related to the mTOR pathway and Proprotein convertase subtilisin/kexin type 9 (PCSK9) after exposure to homoyessotoxin and the laboratory-generated SSA. In contrast, we observed a significant decrease in gene expression in the mTOR pathway and of PCSK9 after exposure to the natural SSA and the mTOR inhibitor, suggesting induction of apoptosis. Our results indicate that marine biogenics in SSAs interact with PCSK9 and the mTOR pathway and can be used in new potential pharmaceutical applications. Overall, our results provide a substantial molecular evidence base for potential beneficial health effects at environmentally relevant concentrations of natural SSAs.

## Introduction

Oceans and seas contain a variety of biogenic or naturally produced molecules that become airborne via sea spray aerosolization^1–3^. In addition to bacteria, which are well-known producers of biogenics, many phytoplankton species also produce a wide range of bioactive molecules such as vitamins, pigments, polyphenolics and phycotoxins, which are potent organic compounds^4,5^. Phycotoxins have primarily been studied in the context of harmful algal blooms, in which they can be present at detrimental concentrations^4,6^. Phycotoxins can be found in seafood and often lead to intoxication or shellfish poisoning due to its consumption^4,7,8^. Furthermore, some of these toxins can cause health effects through their presence in sea spray aerosols. This has been reported for brevetoxins which is a group of toxic cyclic polyethers produced by the dinoflagellate *Karenia brevis* among others^9^. Exposure to aerosolized brevetoxins can lead to respiratory symptoms in humans during algal bloom conditions, particularly in people with asthma^10,11^. The effects of brevetoxins have been well-studied and documented^6,9–11^.

Little attention has, however, been given to other phycotoxins and to their potential effects at the low, environmentally relevant, concentrations in which they may be present in sea spray aerosols (SSAs) during regular environmental conditions^12^. In addition, some of these bioactive molecules (e.g. yessotoxin)^13^ have been targeted for their pharmaceutical or biotechnological potential^14,15^. Yessotoxin, produced by marine dinoflagellates such as *Protoceratium reticulatum*, appears to induce apoptotic cell death through the mammalian target of rapamycin (mTOR) pathway^16^ and seems to inhibit tumor growth^17^. Combined with other unidentified biogenics in the marine environment, these known bioactive molecules could contribute to beneficial health effects in coastal environments. A number of studies highlight several health promoting pathways through which airborne microbiota and biogenics from blue and green environments may have beneficial health effects^18,19^. Airborne microbiota are thought to contribute to a more effective immuno-regulation once inhaled or ingested^18^. Additionally, it was suggested that inhalation of low levels of microbes and parasites reduces inflammation and improves immunoregulation^18,20^. Biogenics, i.e. natural chemicals produced by plants, fungi, phytoplankton species and bacteria^1,3,12^, have been hypothesized to induce positive health effects via the interaction with specific cell signaling pathways such as the mTOR pathway^19^. The mTOR pathway is a key regulator of cell growth and cell proliferation that integrates signals from both the environment (e.g. nutrients) and internal processes (e.g. energy status, growth factors) to regulate several cellular processes including autophagy and energy metabolism^21^. The link between the mTOR pathway and beneficial health effects is supported by a large number of studies^22–26^, demonstrating that inhibition of this cell signaling pathway is associated with health benefits such as anti-cancer and anti-inflammatory effects.

Here, we hypothesize that beneficial health effects of SSAs in coastal environments can be attributed to interactions between marine biogenics such as yessotoxin and the mTOR pathway. To this end, we exposed human epithelial lung cells to extracts of (1) the pure bioactive molecule homoyessotoxin (hYTX), (2) a SSA generated in a laboratory tank inoculated with the homoyessotoxin producing dinoflagellate *Protoceratium reticulatum*^27^, (3) a natural SSA collected at the seashore, and (4) a chemical inhibitor of the mTOR pathway (Torkinib/PP242). In our design, we start from the simplest situation: the exposure to one biogenic molecule (hYTX) as a single substance and extrapolate to a more complex but characterized laboratory generated sample and finally to a black-box environmental mixture (i.e. natural SSA). We used RNA sequencing to characterize the molecular responses. The different treatments, including different dose levels per treatment, allowed us to study a range of conditions, from most realistic, i.e. natural SSA, to the simplest, i.e. a single biogenic molecule (hYTX). With this experimental design, we will address the following research questions: (1) the effects of pure hYTX as shown in previous studies are similar to the effects of a SSA extract generated using a laboratory aerosol tank inoculated with a hYTX producer at the same hYTX dose levels, (2) the effects of a SSA extract generated using a laboratory aerosol tank can be extrapolated to effects of a natural SSA collected at the seashore at more environmentally realistic dose levels and (3) hYTX, a SSA extract generated in the lab and a natural collected SSA extract all interact with the mTOR pathway in human lung cell lines. As such, we aim to provide molecular evidence to support the hypothesis that SSAs are a source of health benefits such as anti-cancer, positive cardiovascular and anti-inflammatory effects.

## Results

**Figure 1:**
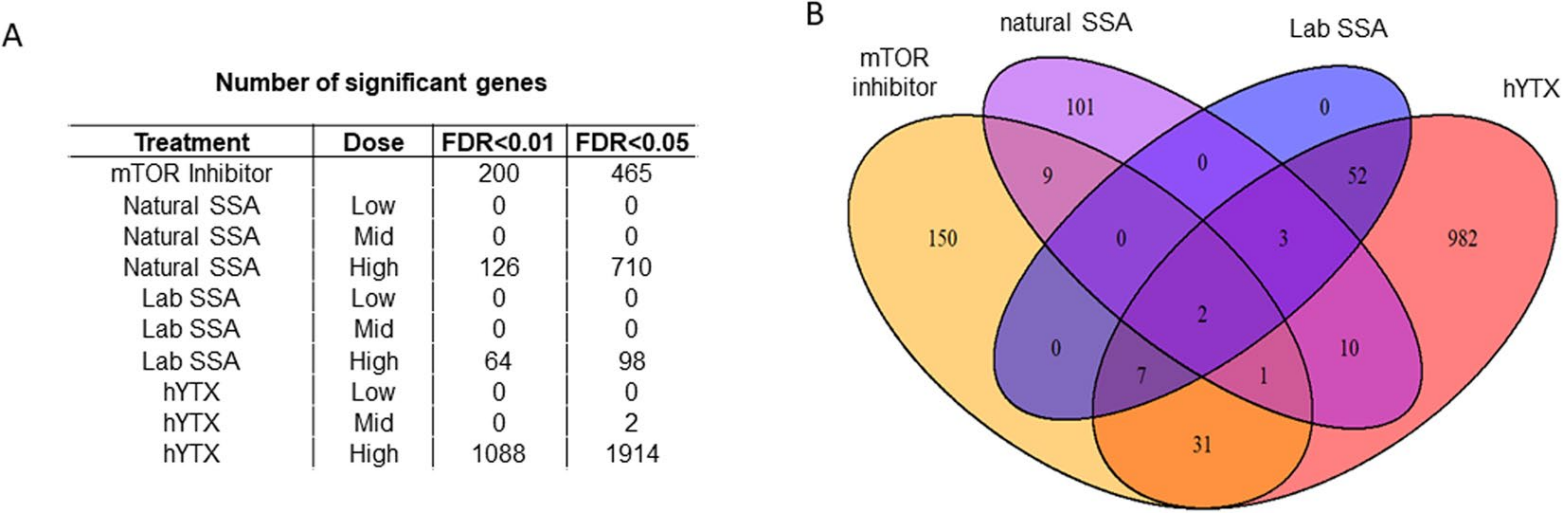
Differential gene expression across treatments. (**A**) Number of significant genes at different false discovery rates (FDR) for the different sea spray aerosols (SSA) treatments and homoyessotoxin (hYTX). (**B**) Venn diagram of shared significant genes across high dose treatments with significant genes at an FDR of 0.01.

We quantified the expression of 16,5654 genes and observed differential expression across all treatments. The highest number of differentially expressed (DE) genes was observed in the pure homoyessotoxin treatment, hereafter referred to as hYTX. We observed a decreasing number of differentially expressed genes in the chemical inhibitor treatment, hereafter referred to as mTOR inhibitor, the natural SSA treatment and the treatment with a SSA generated using a laboratory aerosol tank, hereafter referred to as labSSA. We observed significant DE genes in all treatments at the highest dose levels at false discovery rates (FDR) of 0.01 and 0.05 (Fig. 1A). Given the small difference between the two FDRs, the most conservative FDR was selected for further analysis. We identified two DE genes shared by all high dose level treatments and the mTOR inhibitor (Fig. 1B) and three DE genes shared by all high dose level treatments. The two DE genes shared by all (high dose level) treatments and the mTOR inhibitor (Fig. 1B) were the small integral membrane protein 29 (SMIM 29) and proprotein convertase subtilisin/kexin type 9 (PCSK9). The three genes shared by all high dose treatments but not with the mTOR inhibitor were stearoyl-CoA desaturase (SCD), cytochrome P450 family 1 subfamily B member 1 (CYP1B1) and peptidyl arginine deiminase 3 (PADI3).

We observed a total of 1898 genes with a significant dose response effect across the three treatments (hYTX, lab SSA and natural SSA). Based on a regression analysis and clustering, we found four clusters of dose response patterns. These clusters all show the same trend which consists of a steep dose response curve for hYTX, while the lab SSA and the natural SSA show a slower increase (Fig. S2). A pathway analysis revealed four pathways that were enriched for genes with a significant dose response effect (Table S3). These pathways are the spliceosome, lysosome, steroid biosynthesis and glycogenesis.

### A comparison of the effects of hYTX and the effects of a SSA extract generated in the lab on gene expression

**Figure 2:**
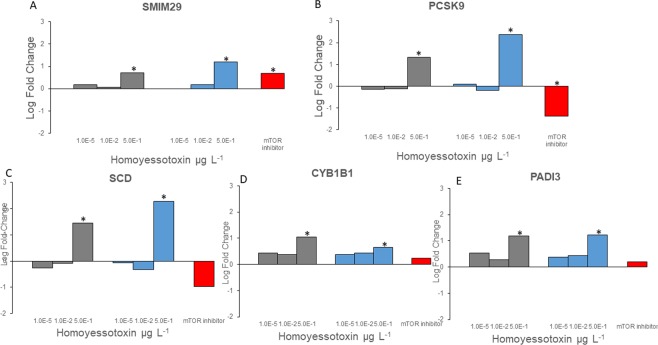
Differential gene expression in the pure homoyessotoxin treatment (hYTX, grey), the laboratory generated sea spray aerosol treatment (Lab SSA, blue) and mTOR inhibitor treatment (red). Log fold change for (**A**) small integral membrane protein 29 (SMIM 29), (**B**) Proprotein convertase subtilisin/kexin type 9 (PCSK9), (**C**) stearoyl-CoA desaturase (SCD), (**D**) cytochrome P450 family 1 subfamily B member 1 (CYP1B1) and (**E**) peptidyl arginine deiminase 3 (PADI3). Stars denote significant gene expression at a false discovery rate of 0.01.

First, we observed that all DE genes regulated by the lab SSA are a subset of the DE genes regulated by hYTX (Fig. 1B). Second, for the five genes shared by the lab SSA, hYTX and the natural SSA (Figs 1B and 2), we see the same dose response pattern for both the hYTX and the lab SSA treatment: increasing gene expression with increasing dose. For SMIM 29, the responses to the highest dose (0.5 µg L^−1^) of both the hYTX and lab SSA treatments were comparable to the increased expression observed for the mTOR inhibitor treatment (Fig. 2A). In contrast, PCSK9 was downregulated by the mTOR inhibitor treatment while it was significantly upregulated at 0.5 µg L^−1^ of hYTX in both the hYTX and lab SSA treatments (Fig. 2B). The three other genes were not significantly regulated by the mTOR inhibitor treatment (Fig. 2C–E).

**Figure 3:**
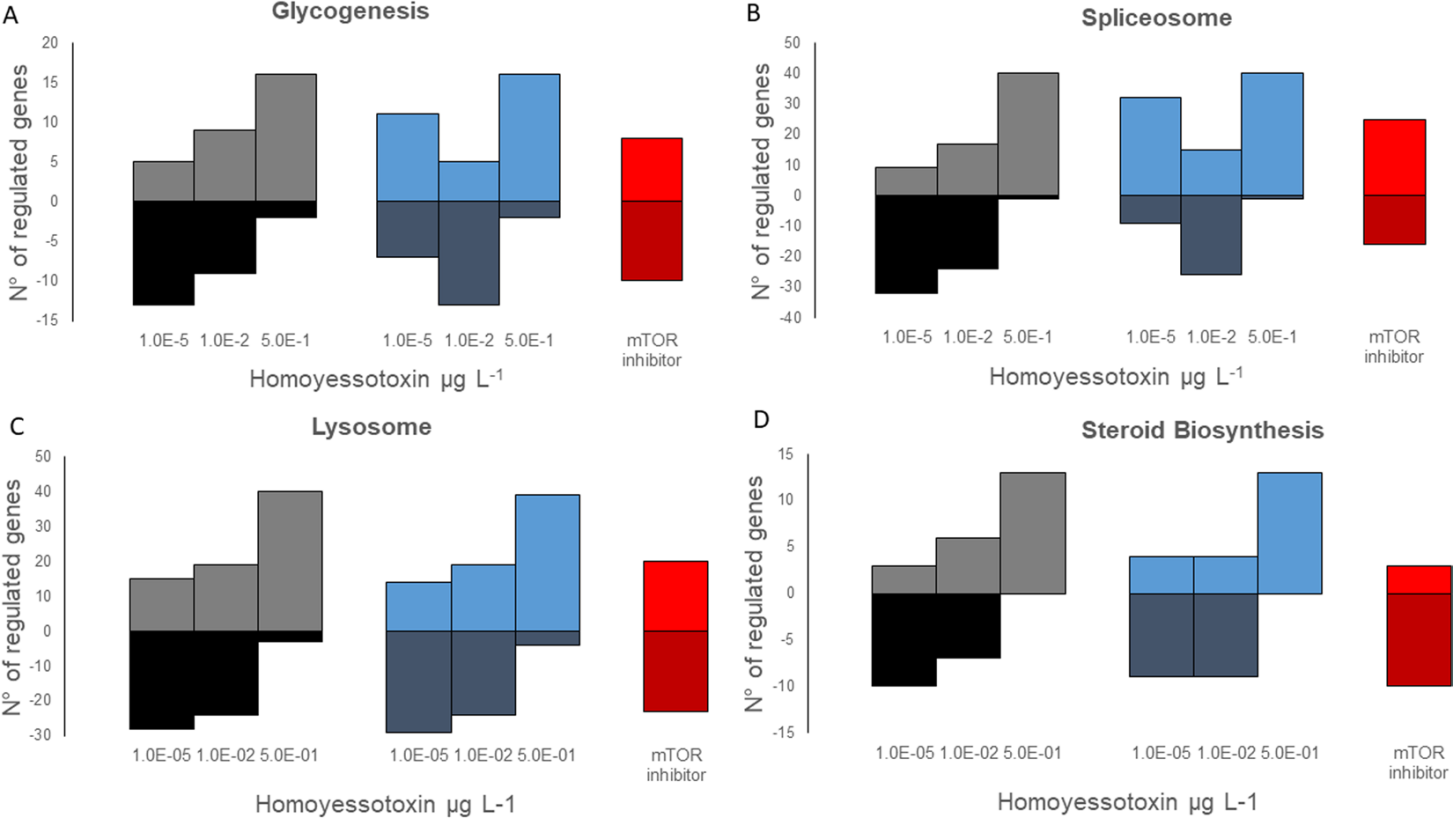
Dose response patterns in significantly affected pathways. Number of significantly upregulated (>0) or downregulated (<0) genes in **(A)** the glycogenesis, **(B)** spliceosome, **(C)** lysosome, **(D**) steroid biosynthesis for the treatments: homoyessotoxin (hYTX, grey), the lab sea spray aerosol (SSA, blue), and the mTOR inhibitor (red).

Third, at the pathway level, we observed an increase in upregulated genes and a decrease in downregulated genes with increasing dose levels of the hYTX treatment for all pathways (Fig. 3). For the lab SSA treatment, this same pattern was observed for the lysosome and steroid biosynthesis but not for the glycogenesis and the spliceosome (Fig. 3). For the glycogenesis and the spliceosome, more upregulated genes in the highest and lowest dose treatment was observed while the mid dose treatment showed more down regulated genes (Fig. 3A,B). For three of the four pathways, the mTOR inhibitor treatment response was similar to the low dose of both the lab SSA and hYTX treatments (Fig. 3A,C,D). For the spliceosome, the response differed (Fig. 3B).

### A comparison of the effects of the SSA extract generated in the lab and the extract of a natural SSA on gene expression

**Figure 4:**
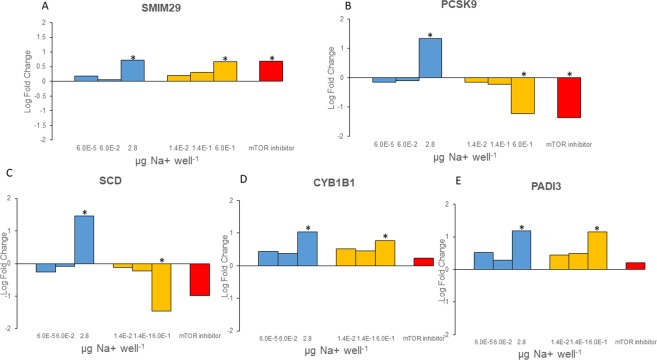
Differential gene expression in the laboratory generated sea spray aerosol treatment (Lab SSA, blue), the natural SSA treatment (yellow) and mTOR inhibitor treatment (red). Log fold change for (**A**) small integral membrane protein 29 (SMIM 29), (**B**) Proprotein convertase subtilisin/kexin type 9 (PCSK9), (**C**) stearoyl-CoA desaturase (SCD), (**D**) cytochrome P450 family 1 subfamily B member 1 (CYP1B1) and (**E**) peptidyl arginine deiminase 3 (PADI3). Stars denote significant gene expression at a false discovery rate of 0.01.

We observed 5 DE genes that were shared between the lab SSA and the natural SSA treatments and these 5 genes were also shared with the pure hYTX treatment (Figs 1B and 4). A direct comparison between the natural and the lab SSA can be made in terms of total mass of the sampled aerosol by using the sodium cation as a proxy for this^28^. The lab SSA dose levels are 2.8 µg Na^+^ well^−1^, 0.06 µg Na^+^ well^−1^ and 0.00006 µg Na^+^ well^−1^ while the natural SSA dose levels, due to the smaller sample size, were 0.6 µg Na^+^ well^−1^, 0.14 µg Na^+^ well^−1^ and 0.014 µg Na^+^ well^−1^ (section 1.2). As such, the high dose natural SSA treatment is almost five times smaller, in terms of aerosol mass, than the high dose lab SSA treatment. This is due to the fact that the levels of the natural SSA were sampled and selected to represent different realistic exposure scenarios whereas the lab SSA levels were sampled and selected to cover the complete dose response curve of hYTX. The low dose natural SSA can therefore be situated between the low and mid dose lab SSA while the mid and high dose natural SSA can be situated between the mid and high dose lab SSA.

For three genes (i.e. SMIM29, CYP1B1, PADI3), the pattern was the same for both treatments with an increased gene expression with increasing dose levels (Fig. 4A,D,E). The two other significantly affected genes (i.e. PCSK9 and SCD), showed a different pattern between these two treatments (Fig. 4B,C). We observed an increased gene expression with increasing dose levels for the lab SSA but a decreased gene expression with increasing dose levels for the natural SSA (Fig. 4). The mTOR inhibitor showed the same response pattern as the highest dose treatment of the natural SSA, which was for mTOR significant for both PCSK9 and SMIM29 (Fig. 4A,B).

**Figure 5:**
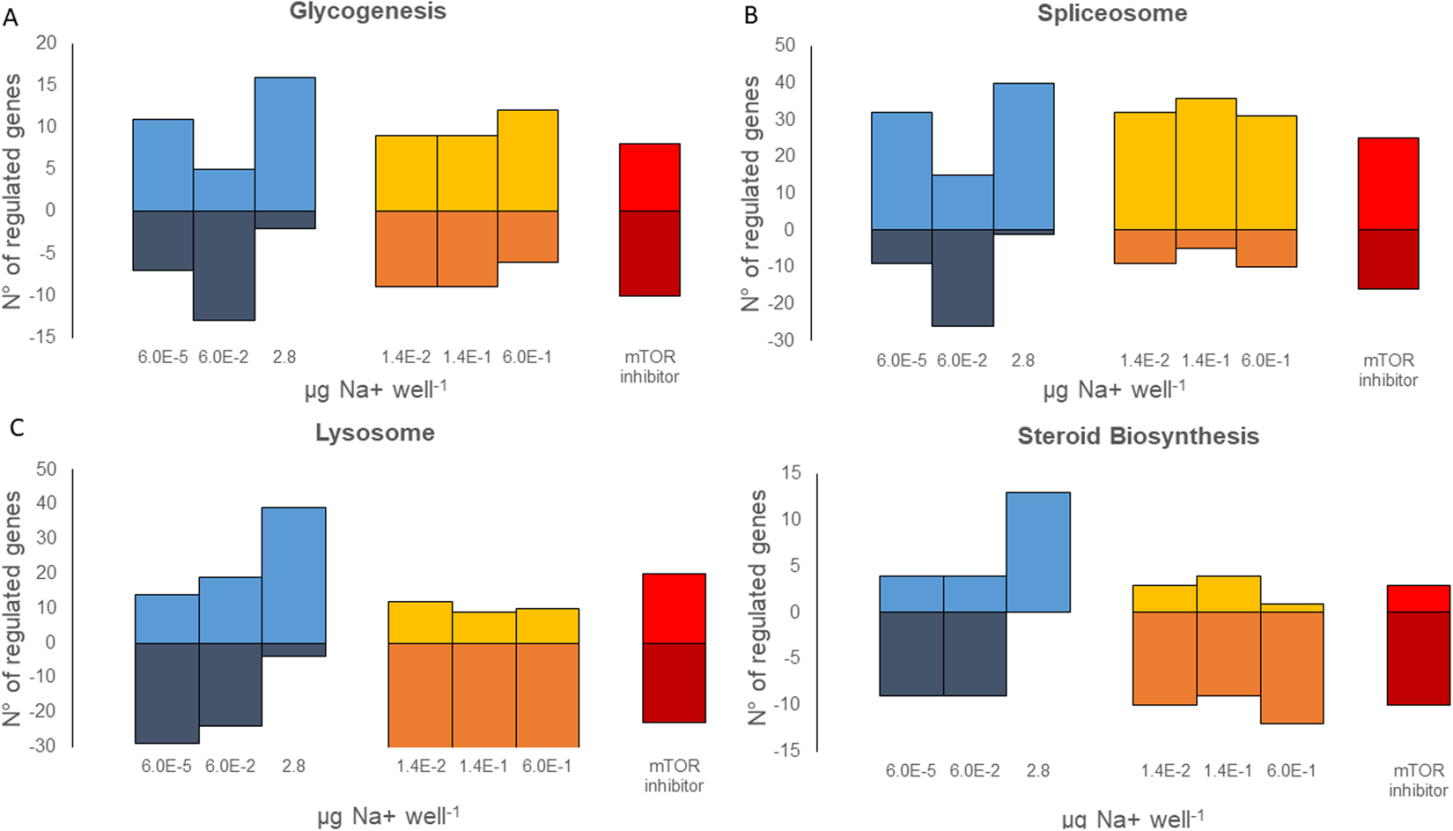
Dose response patterns in significant pathways. Number of significantly upregulated (>0) or downregulated (<0) genes in **(A)** the glycogenesis, **(B)** the spliceosome, **(C)** lysosome, **(D**) steroid biosynthesis for the treatments: lab sea spray aerosol (SSA, blue), natural SSA (yellow) and mTOR inhibitor (red).

At the pathway level, we studied the four pathways with a significant dose response pattern: glycogenesis, lysosome, spliceosome and steroid biosynthesis. Overall, we observed different patterns for the two treatments across the four pathways (Fig. 5). For example, for the steroid biosynthesis, we observed more upregulation with increasing dose levels for the lab SSA and more downregulation with increasing dose levels for the natural SSA. In all pathways, the response of the mTOR inhibitor treatment was comparable to the response of the natural SSA (Fig. 5).

### All treatments interact with the mTOR regulatory pathway

**Figure 6:**
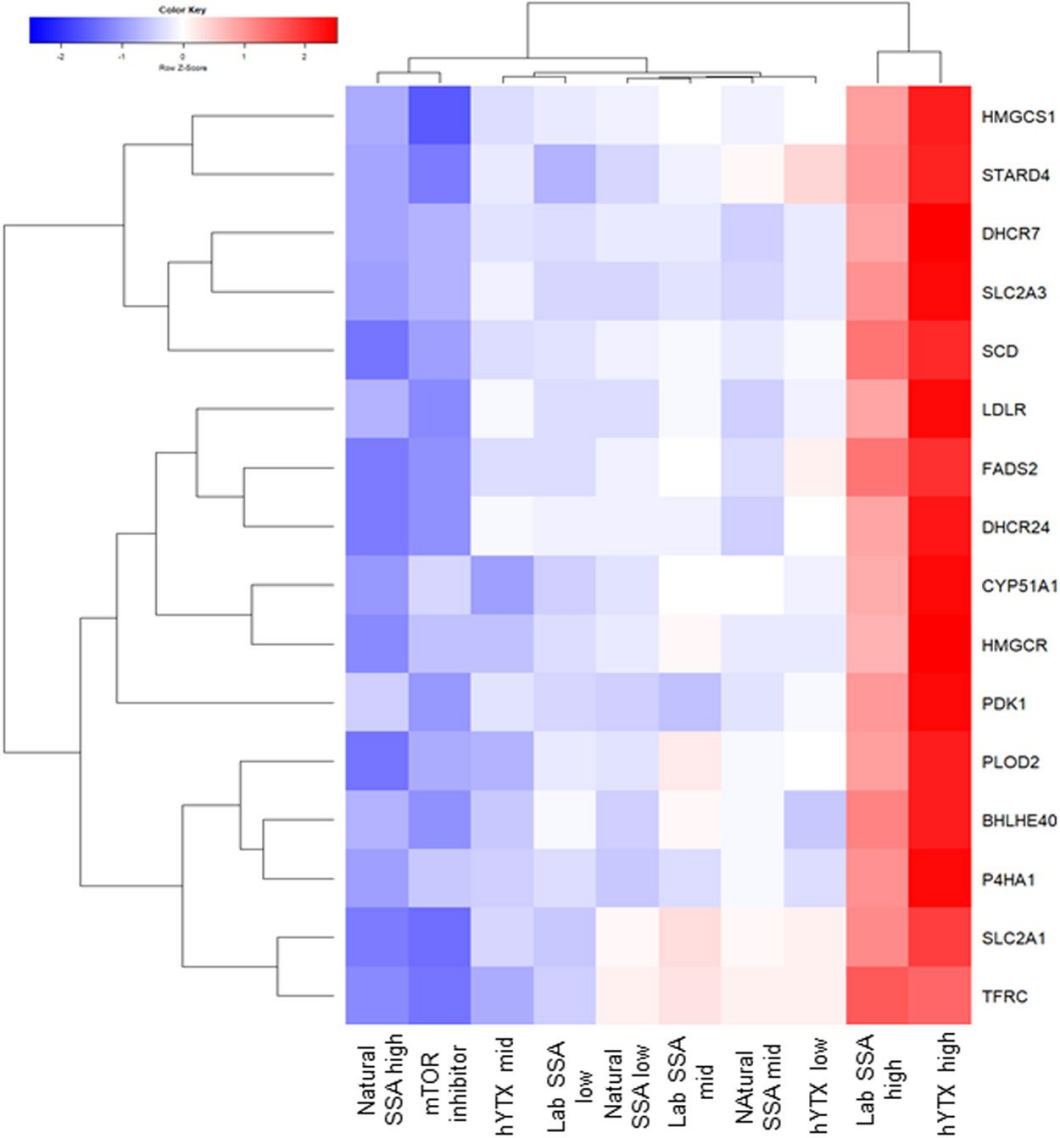
Enrichment of the mTOR Hallmark set. Heatmap for all treatments of the fold changes of genes that contribute significantly to the enrichment score for all three treatments at the highest dose and the mTOR inhibitor. Treatments: chemical inhibitor, homoyessotoxin (hYTX), lab sea spray aerosol (lab SSA) and natural sea spray aerosol (SSA) at high, mid and low doses. For hYTX and the labSSA hYTX levels are: 0.5 µg L^−1^ (high), 0.01 µg L^−1^ (mid), 0.00001 µg L^−1^ (low). For the lab SSA, sodium levels are: 2.8 µg Na^+^ well^−1^(high), 0.06 µg Na^+^ well^−1^(mid), and 0.00006 µg Na^+^ well^−1^(low), and for the natural SSA, sodium levels are: 0.6 µg Na^+^ well^−1^(high), 0.14 µg Na^+^ well^−1^ (mid) and 0.014 µg Na^+^ well^−1^(low).

Here, the genes of the mTOR pathway (as defined by the KEGG database)^29^ and a hallmark set of genes upregulated upon activation of the mTORC1 complex (as defined by the molecular signature databases)^30^ were used to evaluate potential effects of the treatments on the mTOR pathway. No enrichment of significantly expressed genes of the mTOR pathway (as defined by the KEGG database) was observed in any of the treatments. However, individual genes of the mTOR pathway (as defined by the KEGG database), were significantly regulated in different high dose treatments, with the exception of the lab SSA for which no genes were differentially expressed (Table S1). Taking a closer look at the hallmark mTORC1 set, we observed that the gene expression patterns differed across treatments (Fig. S1). Hierarchical clustering of these patterns indicated that differentially expressed genes were in general regulated in the opposite direction for hYTX and the lab SSA versus the natural SSA and the chemical inhibitor (Fig. S1). This pattern is even more prominent when focusing on the genes that contribute significantly (FDR < 0.05) to the enrichment score in the hallmark set for all 4 treatments (Fig. 6). This group of 17 genes showed completely opposite regulation patterns in the high dose hYTX and high dose lab SSA versus the high dose natural SSA and the chemical inhibitor (Fig. 6). The first group showed increased expression or upregulation of hallmark genes, confirmed by a significant positive enrichment score of 2.14 and 1.92 for the pure hYTX and lab SSA treatments respectively (Table S2). In contrast, the chemical inhibitor and high dose natural SSA treatments showed an inhibition of expression or downregulation of the hallmark genes, reflected by a negative enrichment score of −2.32 and −1.24 for the mTOR inhibitor and the natural lab SSA treatment respectively (Table S2).

## Discussion

It has been postulated that biogenic molecules in SSAs lead to beneficial health effects in humans through interactions with the mTOR pathway^19^. Here, we report the effects of a pure biogenic molecule hYTX, the extract of a lab generated SSA (containing hYTX from an algal producer) and the extract of a natural SSA sampled from the environment on human lung cells.

We observed a high overlap between the effects of the pure hYTX and the lab SSA treatments at the gene and pathway level. This suggests that the effects of the lab SSA are most likely comparable to effects of a diluted hYTX treatment. Or, in other words, the effects of the lab SSA containing hYTX from an algal producer are weaker than the effects of the pure hYTX treatment despite containing the same amount of hYTX. This suggest that (1) the lab SSA may contain additional molecules which interact with hYTX leading to weaker effects or that (2) the lab SSA may contain hYTX or YTX analogues or metabolites with potential weaker effects that compete with hYTX for molecular binding sites and uptake. Both assumptions suggest a lower bioavailability of pure hYTX, potentially leading to a lower actual dose.

Interestingly, the effects of the natural SSA at the gene and pathway level closely resemble the effects of the mTOR inhibitor, but contrast with the effects of hYTX and the lab SSA. The differences between these two treatment clusters highlight that while all treatments target the mTOR pathway, their effects are opposite. This suggests that the natural SSA is a complex mixture of biogenics interacting with the mTOR pathway leading to different expression patterns of the same pathway than the lab SSA. Literature reports only briefly on the organic composition of SSAs, but suggests a large diversity in biogenic compounds^31,32^. The similarities in regulation of the mTOR pathway between the natural SSA and the chemical inhibitor suggest that natural SSAs contain molecules that cause similar effects on the mTOR pathway as the chemical inhibitor. The differences between the lab SSA and the natural SSA could be related to the differences in doses or the presence of other or additional unknown molecules. The high dose treatment for both hYTX and the lab SSA of 0.5 µg hYTX liter^−1^ is an extreme case scenario, reflecting concentrations in water during harmful algal blooms (supportive information 1.2). The environmental (background) concentrations of hYTX in water and air have not been previously reported but are expected to lie between the low and mid dose levels based on estimates of cell counts of hYTX producers and hYTX production per cell (supportive information 1.2). As such, it is clear that while the exact regulation of genes and pathways differs between lab SSA and natural SSA extracts, both samples significantly interact with the mTOR pathway. Furthermore, the effects of the natural SSA extract are similar to the effects of the chemical mTOR inhibitor suggesting beneficial health effects at environmentally relevant concentrations.

We observed two genes significantly regulated by all treatments and by the mTOR inhibitor. The first gene was the small integral membrane protein 29 (SMIM 29). Little functional information on this protein is available, although it is ubiquitously expressed in at least 25 tissues^33^. The other gene is proprotein convertase subtilisin/kexin type 9 (PCSK9), primarily involved in lipid homeostasis and apoptosis^34^. PCSK9 is thought to have two major functions: (1) maintenance of lipid homeostasis by the regulation of low-density lipoprotein receptors and (2) the regulation of neural apoptosis^34^. In general, the overexpression or upregulation of PCSK9 is associated with the dysregulation of pathways involved in the cell cycle, inflammation and apoptosis while the inhibition or downregulation of PCSK9 in carcinogenic lung cells has been associated with apoptosis of these cell lines^34^. In mouse, a similar pattern has been observed^35^. Upregulation of PCSK9 was associated with multi-organ pathology and inflammation while PCSK9 downregulation was associated with protection against inflammation, organ pathology and systemic bacterial dissemation^35^. These findings in literature together with our results, (i.e. downregulation of PCSK9 in the mTOR inhibitor and the natural SSA treatments) suggest beneficial health effects of natural SSAs through the apoptosis of lung cancer cells. Based on the results provided here on PCSK9, we propose that SSAs contain molecules with significant pharmaceutical potential in targeting PCSK9^36^.

We also observed three genes that were significantly regulated by all treatments but not by the mTOR inhibitor: SCD, PADI3 and CYP1B1. The pattern of SCD was comparable to that of PCSK9 while the patterns of PADI3 and CYP1B1 were comparable to the pattern of SMIM29. This can be attributed to the functions of SCD and PCSK9, as both are involved in lipid biosynthesis. Furthermore, research has already indicated links between the mTOR pathway and the lipid homeostasis^37^, including the effects on SCD and other genes after exposure to mTOR inhibitors^37^. Evidence points to the sterol regulatory element binding transcription factor 1 (SREBF1) through which the regulation of lipogenesis by mTOR is achieved^37^. This gene was significantly regulated by the natural SSA treatment, but not by any of the other treatments. CYP1B1 is commonly involved in the metabolism of xenobiotics and could play a role in metabolizing some of the biogenic molecules. Literature has also reported a relation between CYP1B1 and SCD in lipid homeostasis in liver cells^38^, although the extent of this relation in lung cells remains unclear. Here we observed an increase in expression of CYP1B1 in all treatments. Overexpression of CYP1B1 has also been reported in lung cell lines through the aryl hydrocarbon receptor^39^, but no significant effects for this receptor were observed in any treatment of our study (Table S4). This suggests that the overexpression of CYP1B1 is more likely related to the regulation of SCD. In addition, PADI3 was also upregulated in all three high dose treatments (hYTX, lab SSA and natural SSA). PADI3 is generally not expressed in lung cells^33^ and is primarily expressed in epidermis cells and keratinocytes^40^. Its function in lung cell lines remains unclear.

At the pathway level, we observed differential expression of genes linked to the mTOR pathway in all three high dose treatments (natural SSA, lab SSA, and hYTX). Our results also indicated significant effects on the mTOR pathway, but the effects and the potential beneficial health effects differ across treatments. Most likely, the effects on these genes are caused by the primary effects on the mTOR pathway. Furthermore, for three genes, these effects while linked to the mTOR pathway, are not observed with the mTOR inhibitor. This suggests that the effects of these experimental treatments (natural SSA, lab SSA, and hYTX) extend beyond the inhibition of mTOR but are related to or initiated by the effects on the mTOR pathway.

We observed four pathways that were significantly affected by the different treatments: glycogenesis, lysosome, spliceosome and steroid biosynthesis. For the steroid biosynthesis, these results are not surprising given the links that have already been discussed above between mTOR and lipid biosynthesis. In addition to steroid biosynthesis, the lysosome and glycogenesis also have links to mTOR. The inhibition of the mTOR pathway is known to activate protein degradation and autophagy through among others the lysosome^41,42^. The spliceosome has been proposed as a therapeutic target in cancer cells to inhibit mTOR, which leads to autophagy^43^. Specifically, depletion of small nuclear ribonucleoprotein polypeptide E (SNRPE) led to reduced cell viability in lung cancer cell lines. Here, we observed in addition to dose response effects for the spliceosome, a significant downregulation of SNRPE in the highest hYTX treatment but not in any of the other treatments (Table S5). Overall, the pathways with significant dose response effects can all be indirectly linked to the mTOR pathway, suggesting that the effects here are a consequence of the effects on the mTOR pathway, which most likely induces a cascade of events and interactions with other pathways.

**Figure 7:**
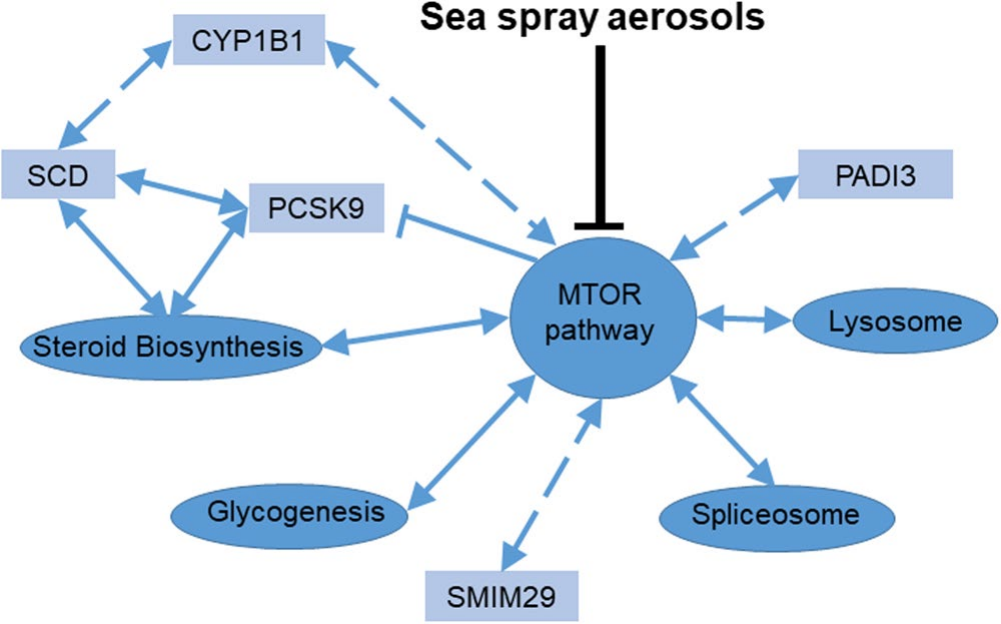
Molecular effects of marine aerosolized biogenics. A schematic representation of the molecular effects of sea spray aerosols observed within this study. Pathways are represented by ellipses, genes are represented by rectangles. Solid blue arrows represent interactions with a solid evidence base, dashed arrows represent hypothetical interactions observed, ⊢ represent inhibition.

Overall, the results at the gene level and at the pathway level highlight that the effects are primarily mediated or linked through the mTOR pathway supporting the biogenesis hypothesis postulated by Moore^19^ that marine airborne biogenics interact with the mTOR pathway leading to health benefits. All treatments significantly affected the mTOR pathway, but we observed differences in the direction of the regulation of this pathway (Fig. 7). Furthermore, significant genes and enriched pathways across treatments all interact with mTOR, indicating that marine biogenics trigger a cascade of events through interaction with the mTOR pathway (Fig. 7). Thus, the effects of marine airborne biogenics are not limited to the mTOR pathway but include a cascade of genes and pathways involved in different metabolic processes (e.g. steroid biosynthesis, lysosome) with key links to mTOR (Fig. 7).

## Methods

### Culturing of A549 cells

Adenocarcinoma alveolar basal cell lines (A549) were maintained in Dulbecco’s Modified Eagle Medium (DMEM), supplemented with 10% fetal bovine serum and 100 units.mL^−1^ penicillin-streptomycin at 37 °C, 5% CO_2_ and >95% relative humidity. Confluent cell cultures (after 2–3 days) were passaged via trypsination (0.5% trypsin-EDTA) and split in a ratio of 1:6.

### Experimental procedure

Confluent cell cultures were trypsinized and transferred in 3 mL fresh DMEM to Nunc 6-well multiplates at a density of 320,000 cells.well^−1^. After seeding, cells were incubated for 10 hours at 37 °C, 5% CO_2_ and >95% relative humidity to stimulate growth and adherence to the surface. Then, cells were subjected to one of five treatments and were then incubated for another 43 hours at identical conditions prior to RNA extraction. The five treatments included (1) unexposed cell lines as a negative control, (2) an extract of a natural SSA sample from the seashore, (3) an extract of a laboratory generated SSA, (4) homoyessotoxin, (5) a chemical inhibitor of the mTOR pathway, i.e. Torkinib or PP242 (LC Laboratories), as a positive control. The negative control treatment also contained 2% methanol to exclude a solvent effect as all other treatments were extracted, diluted or dissolved in methanol. The chemical inhibitor treatment consisted of 0.3 µM of Torkinib or PP242. All aerosol samples were collected on a Whatman QM-A quartz microfiber filter using a membrane vacuum pump at the constant flow rate of 10 L min^−1^. The natural sea spray aerosol sample was collected at the waterline close to Ostend, Belgium (51°14′27″N, 2°56′10″E) by sampling for 46 minutes at a flow of 10 L min^−1^, which corresponds to the minute ventilation of an average human in rest (9–10 L min^−1^)^44,45^. During sampling, the wind direction was 0.7 ± 3.1° (North) and wind speed was 15.0 ± 0.6 m s^−1^, indicating white cap SSA production. The detailed sampling and extraction procedure is described in supportive information 1.1. The lab SSA was obtained by inoculating a marine aerosol reference tank^46^ with 10^6^ cells L^−1^ of *Protoceratium reticulatum*, a hYTX producer (SCCAP K-1474), and by collecting the generated SSA at a flow of 10 L min^−1^ for 16 hours to obtain sufficient material for further experiments and analysis. The detailed procedure is described in supportive information 1.1. Filters of the natural SSA and lab SSA were extracted following the same methanol extraction procedure. Certified reference material of hYTX was commercially obtained (National Research Council Canada) as a liquid with a concentration of 5 µM hYTX dissolved in methanol. This reference material was further diluted in methanol to obtain the following dose levels: 0.5 µg L^−1^ (high), 0.01 µg L^−1^ (mid), 0.00001 µg L^−1^ (low). Concentrations of hYTX in the lab SSA were measured using ultra-high-performance liquid chromatography high-resolution Orbitrap mass spectrometry following procedures as reported by Orellana *et al*.^47^. To allow an optimal comparison between the hYTX treatment and the lab SSA, the lab SSA dose levels were determined based on the measured hYTX in these samples and the same dose levels as the hYTX treatment were selected (0.5 µg L^−1^ (high), 0.01 µg L^−1^ (mid), 0.00001 µg L^−1^ (low)). For the natural SSA, low, mid and high doses were determined by comparing the total alveolar surface of human lungs with the cell surface available in a single well (9.6 cm²) and comparing the sample collection duration (46 min) and experimental exposure duration (43 h), see supportive information 1.2. We selected a low dose that represents the same exposure as the amount of inhaled SSA during the sampling period at the seashore but extended over a 43 h exposure period and normalized to the cell surface in a single well (detailed calculations are reported in supportive information, section 1.2). The mid and high dose represent a 10x and 40x concentration of the low dose level. These levels were specifically chosen to adhere to environmentally realistic (background) concentrations. The mid dose level (10x concentration) was based on the hypothesis of increased minute ventilation during physical exercise which is reported to vary between 70–100 L min^−1^ for both continuous and intermittent exercise^45,48,49^. The high dose level (40x concentration) was selected based on the hypothesis of increased aerosolization (i.e. improved wind conditions) as well as activities at the shore line or at sea (e.g. swimming, sailing, windsurfing, ...). The detailed procedure is described in the supportive information, section 1.2.

### RNA extraction, library preparation and sequencing

RNA was extracted using the Qiagen RNEasy kit following the manufacturer’s instructions including DNAse digestion. After RNA extraction, the concentration and quality of the total extracted RNA was checked by using the ‘Quant-it ribogreen RNA assay’ (Life Technologies, Grand Island, NY, USA) and the RNA 6000 nano chip (Agilent Technologies, Santa Clara, CA, USA), respectively. Subsequently, 250 ng of RNA was used to perform an Illumina sequencing library preparation using the QuantSeq 3′ mRNA-Seq Library Prep Kits (Lexogen, Vienna, Austria) according to manufacturer’s protocol. During library preparation 14 PCR cycles were used. Libraries were quantified by qPCR, according to Illumina’s protocol ‘Sequencing Library qPCR Quantification protocol guide’, version February 2011. A high sensitivity DNA chip (Agilent Technologies, Santa Clara, CA, US) was used to control the library’s size distribution and quality. Sequencing was performed on a high throughput Illumina NextSeq500 flow cell generating 75 bp single reads.

### Data analysis

Per sample, on average 7.5 × 10^6^ ± 1.6 × 10^6^ reads were generated. First, these reads were trimmed using cutadapt^50^ version 1.15 to remove the “QuantSEQ FWD” adaptor sequence. The trimmed reads were mapped against the Homo sapiens GRCh38.89 reference genome using STAR^51^ version 2.5.3a. The RSEM^52^ software, version 1.3.0, was used to generate the count tables. Differential gene expression analysis between groups of samples was performed using edgeR^53^. Genes with less than 1 cpm in less than 4 samples were discarded, resulting in 16,546 quantifiable genes. Read counts were normalized using trimmed mean of M-values (TMM) followed by a pairwise comparison of treatments with the negative and positive control using an exact test^53^. Significantly differentially expressed (DE) genes were called at a false discovery rate of 0.01. Significant enrichment of KEGG pathways^29^ with DE genes was done using a fisher test and called at an adjusted p-value level of 0.01. Benjamini-Hochberg adjustment was used to account for multiple testing. Gene set enrichment analysis (GSEA) was conducted to detect enrichment in hallmark gene sets and genetic and chemical perturbations gene sets of the molecular signature database^30^. Enriched gene sets were identified at a false discovery rate of 0.01. A dose response analysis was performed with the maSigPro^54^ R package for each of the three treatments of algal toxins. In a first step a general linear model was built with the 3 treatments, 3 concentrations and the square of each of concentration. Statistical testing was done using the log-likelihood ratio statistic. Genes with a FDR < 0.05 were considered significantly differential. In a second step, for each significant differentially expressed gene, an optimized regression model was created using stepwise backward regression. Exclusion of the quadratic term from the model was performed using a regression ANOVA, testing if the regression coefficients differ from 0 at a significance level of 0.05. Afterwards the goodness of fit, R², of each optimized regression model was computed. Genes with a goodness of fit greater than 0.8 were used in a hierarchical cluster analysis based on the correlation between the regression models of the genes.

## Supplementary information

Supplementary information

Supplementary Dataset Table S3

## Data Availability

Raw and processed sequencing reads are deposited in GEO and available under accession number: GSE113144.
