# Supplementary material for: Marine biogenics in sea spray aerosols interact with the mTOR signaling pathway
Source: Sci Rep. 2019 Jan 24;9:675. doi: 10.1038/s41598-018-36866-3 (PMC6345880; doi:10.1038/s41598-018-36866-3)
Supplement: Supplementary file 1 — Supplementary information [file 41598_2018_36866_MOESM1_ESM.pdf]

Supportive Information

Marine biogenics in sea spray aerosols interact with the mTOR signaling pathway.

Jana Asselman[1§*], Emmanuel Van Acker[1§*], Maarten De Rijcke[2], Laurentijn Tilleman[3], Filip Van Nieuwerburgh[3], Jan Mees[2], Karel A.C. De Schamphelaere[1], Colin R. Janssen[1]

[1]Laboratory of Environmental Toxicology and Aquatic Ecology, Environmental Toxicology Unit - GhEnToxLab, Ghent University, Campus Coupure, Coupure Links 653, Building F – 2nd Floor, Gent, Belgium, www.ecotox.ugent.be

[2]Flanders Marine Institute (VLIZ), InnovOcean site, Wandelaarkaai 7, 8400 Oostende, Belgium, www.vliz.be

3 Laboratory for Pharmaceutical Biotechnology, Faculty of Pharmaceutical Sciences, Ghent University, Campus UZ, Ottergemse Steenweg 460, 9000 Ghent, Belgium, www.ugent.be/fw/pharmaceutics/pharmbiotech/

[§]These authors contributed equally

*Corresponding authors: Jana.Asselman@UGent.be & Emmanuel.VanAcker@UGent.be

Laboratory of Environmental Toxicology and Aquatic Ecology, UGent

Campus Coupure, Coupure Links 653, Building F – 2nd Floor,

9000 Gent, Belgium

1. Methods

1.1 Extraction of sea spray aerosols from membrane filters.

Sea spray aerosols (SSAs) were extracted from membrane filters following the protocol described in Baelus (2016). In brief, filter were cut in two equal pieces. One of these halves was eluted with methanol followed by sonication. These elution steps were repeated a second time. Then, the combined eluent was filtered over a 0.2 µm PTFE filter. This was followed by evaporation of the sample under a gentle nitrogen gas stream until ±250 µL remained. Half of this extract (mass based) was stored at -20°C for further experimental use, with the other half the hYTX concentration was determined using the analytical techniques described in Orellana et al. (2014)[1]. The other corresponding half of the filter was eluted with 0.14M HNO_3 followed by a vortex, sonication and centrifugation step. These elution steps were repeated a second and third time. Then, the joined eluent was filtered over a 0.45 µm Supor filter and analysed for the Na^+ content using inductively coupled plasma optical emission spectrometry (ICP-OES).

The natural SSA extract was not analyzed for phycotoxins. Via measurements of similar samples from the same time period (July 2017), however, we can conclude that no phycotoxins in this rather small sample would have exceeded the limits of detection.

1.2 Determination of dose levels for different treatments

Currently, literature reports homoyessotoxin concentrations in shellfish and other marine organisms but not in water nor in SSAs. As such, we made certain assumptions to decide on the exposure dose levels for homoyessotoxin and the laboratory generated sea spray aerosol. First, reported cell densities[2,3] of *Protoceratium reticulatum* range between 400 – 700 cells/L in the marine environment. Second, reported homoyessotoxin concentrations[4,5] range between 28.6 to 33.6 pg per cell of *Protoceratium reticulatum*. Based on these reports, we estimated a concentration of 0.01 µg L^{-1} as the mid dose. We determined the high dose as 0.5 µg L^{-1} (x50) as an extreme case scenario and 0.00001 µg L^{-1} (1/1000) as the low dose as background cell densities were reported as at most 10 cells per liter[2] . This would allow us to cover the full width of the dose response curve.

For the natural SSA sample, we aimed at selecting environmentally relevant doses. Hence, we sampled for 45 minutes at the sea shore on a windy day at a volume of 10 L min^{-1}, equivalent to the minute ventilation in rest for an average person[6,7]. Given that the multiwell plates only have a surface of 9.6 cm^2 whereas the total lung surface is reported to be between 35-140m^2 depending on body size, measurement technique and inhalation or expiration[8]. Given the variation in reported size for the total lung surface, we selected 40m^2 as average epithelial alveolar surface. We needed to account for the reduction in surface size as otherwise the amount of aerosols per surface would be larger than in reality. In addition, we also accounted for the exposure duration. As a result, during our sampling, we collected 460L of air, which would theoretically be taken up by the entire alveolar surface (40 m^2). Hence, we calculated the amount of air taken up by 9.6 cm^2 cells, which is 0.011 liter. This amount of air is inhaled in a period of 46 min, while the experimental exposure will last 43 hours. Hence, we calculated the amount of air that would hypothetically be inhaled in exposure period of 43 hours, which is 0.619L. As such, this is relatively 0.26% of the filter extract. This level was selected as the low dose level. We then then determined the mid and high dose treatment by using a factor 10 (2.6%) and 40 (10.8%) relative to the low dose. The factor 10 accounts for increased breathing during exercise and activity while the factor 40 additionally accounts for variation in weather conditions (e.g. more favorable aerosolization conditions due to subsequent windy days, giving higher waves and a subsequent higher SSA production).

1.3 Quantification of sodium in SSA as a proxy for total SSA mass.

The sodium in lab and natural SSA samples was quantified using one half of the quartz filter containing the aerosol. Five mL of 0.14 M HNO3 was added to this half of the filter in a falcon tube

and vortexed for 20 seconds. The sample was then sonicated for 5 minutes after which the supernatant was transferred to a new 15 mL falcon tube. Then, 0.14 M HNO3 was added to make up a total volume of 15 mL, followed by vortexing the sample and centrifuging the sample for 5 minutes at 4000 RCF. The supernatant was transferred to a 20 mL syringe equipped with a Supor 0.45 µm filter, which was rinsed with ± 2mL supernatants (=waste). Then, ± 8 mL of the supernatant was transferred in an AAS-tube and analyzed with using inductively coupled plasma optical emission spectrometry (ICP-OES).

Table S1: Enrichment scores (ES), normalized enrichment scores (NES) and the false discovery rate (FDR) for gene set enrichment analysis of the mTOR hallmark gene set for all treatments.

	Natural sea spray aerosol			Laboratory sea spray aerosol			Homoyessotoxin			mTOR Inhibitor
	High	Mid	Low	High	Mid	Low	High	Mid	Low	
ES	-0.25	0.31	0.16	0.39	-0.18	-0.2	0.46	-0.35	-0.26	-0.48
NES	-1.24	1.48	0.83	1.92	-0.83	-0.92	2.14	-1.7	-1.2	-2.32
FDR	0.20	0.04	1	0.001	1	0.9	0	0.006	0.38	0

Table S2: Genes, assigned to the mTOR pathway based on KEGG pathway annotations, with a false discovery rate (FDR) <0.01 in at least 1 treatment. The false discovery rates and log2 fold changes are reported for all high dose treatments (the natural and laboratory sea spray aerosol (SSA), homoyessotoxin (hYTX)) and the mTOR inhibitor. Significances are highlighted in bold

	Natural SSA		Lab SSA		hYTX		mTOR inhibitor	
Ensembl Gene ID	FC	FDR	FC	FDR	FC	FDR	FC	FDR
ENSG00000167965	**0.85**	**7.73 e-03**	0.29	0.78	0.29	0.78	0.1	0.53
ENSG00000168209	-0.20	0.70	-0.28	0.84	**1.19**	**4.49 e-06**	**-0.96**	**7.24 e-03**
ENSG00000173511	0.16	0.71	0.34	0.70	**0.98**	**2.85 e-05**	0.24	0.64
ENSG00000171608	0.23	0.40	0.03	1.00	**0.58**	**2.95 e-03**	-0.07	0.94
ENSG00000063046	0.25	0.40	0.04	0.94	-0.07	0.94	**0.88**	**4.61 e-05**
ENSG00000117461	0.12	0.80	0.25	0.84	0.21	0.61	**-1.21**	**1.14 e-04**

Table S3 Kegg pathways and their corresponding adjusted p-value for Fisher enrichment test of genes with a significant dose response effect (false discovery rate (FDR) <0.01)): see separate excel file.

Table S4: Log2 Fold change (FC) and false discovery rates (FDR) for the aryl hydrocarbon receptor (AHR) in all treatments. Significances are highlighted in bold.

Treatment	Dose	FC	FDR
Natural sea spray aerosol	Low	-0.19	1.00
Natural sea spray aerosol	Mid	-0.18	1.00
Natural sea spray aerosol	High	-0.13	0.86
Laboratory sea spray aerosol	Low	-0.06	1.00
Laboratory sea spray aerosol	Mid	0.16	1.00
Laboratory sea spray aerosol	High	-0.65	0.55
Homoyessotoxin	Low	0.11	1.00
Homoyessotoxin	Mid	-0.17	0.89
Homoyessotoxin	High	0.25	0.67
mTOR inhibitor	--	0.17	0.88

Table S5: Log2 Fold change (FC) and false discovery rates (FDR) for the small nuclear ribonucleoprotein polypeptide E (SNRPE) in all treatments. Significances are highlighted in bold.

Treatment	Dose	FC	FDR
Natural sea spray aerosol	Low	0.01	1.00
Natural sea spray aerosol	Mid	0.12	1.00
Natural sea spray aerosol	High	0.01	1.00
Laboratory sea spray aerosol	Low	-0.03	0.90
Laboratory sea spray aerosol	Mid	-0.13	1.00
Laboratory sea spray aerosol	High	-0.35	0.57
Homoyessotoxin	Low	-0.27	1.00
Homoyessotoxin	Mid	-0.25	0.54
Homoyessotoxin	High	-0.83	**4.58 e-05**
mTOR inhibitor	--	0.14	0.76

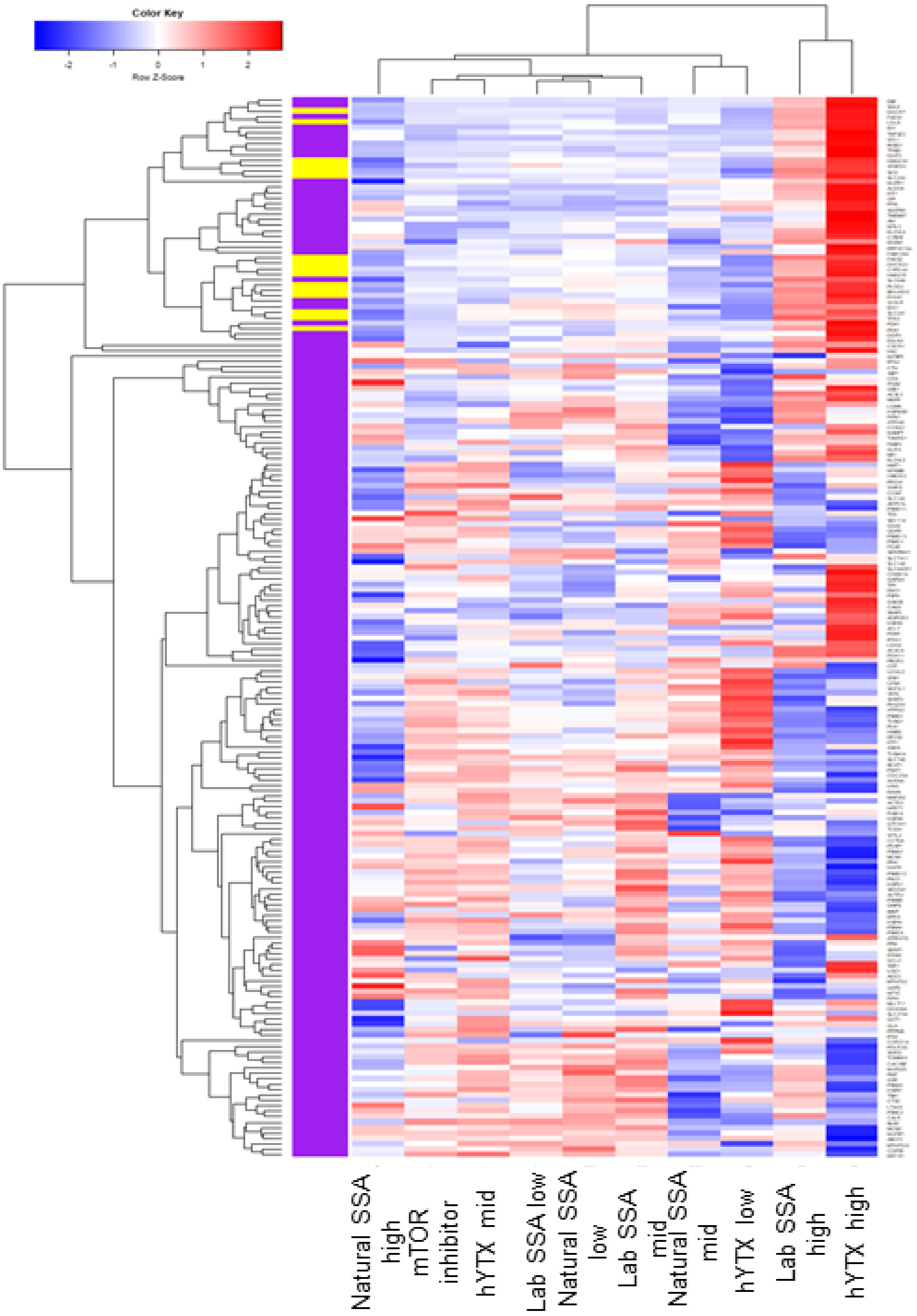

Figure S1: Heatmap of the mTOR Hallmark set for all treatments (the natural and laboratory sea spray aerosol (SSA), homoyessotoxin (hYTX)) for all dose levels and the chemical inhibitor. Yellow band distinguishes genes enriched in all three high dose treatments and the chemical inhibitor from the other hallmark genes (purple).

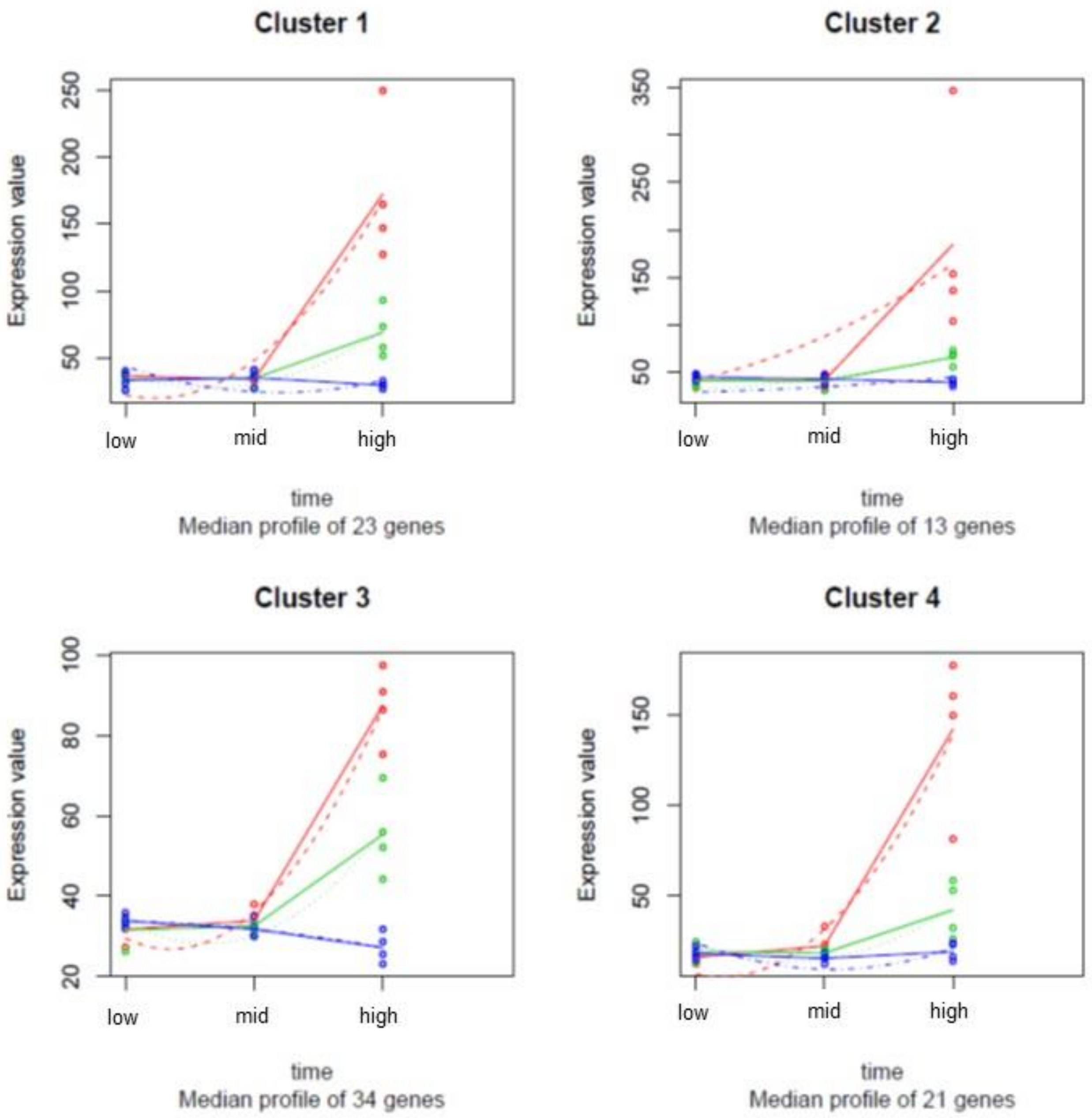

Figure S2: Median gene expression profiles for the different clusters plotted per treatment. Red represents the homoyessotoxin treatment, green represents the lab sea spray aerosol treatment and blue represents the natural sea spray aerosol treatments. Points are median values connected by solid lines, dotted lines are the regression models. Expression values were normalized counts.

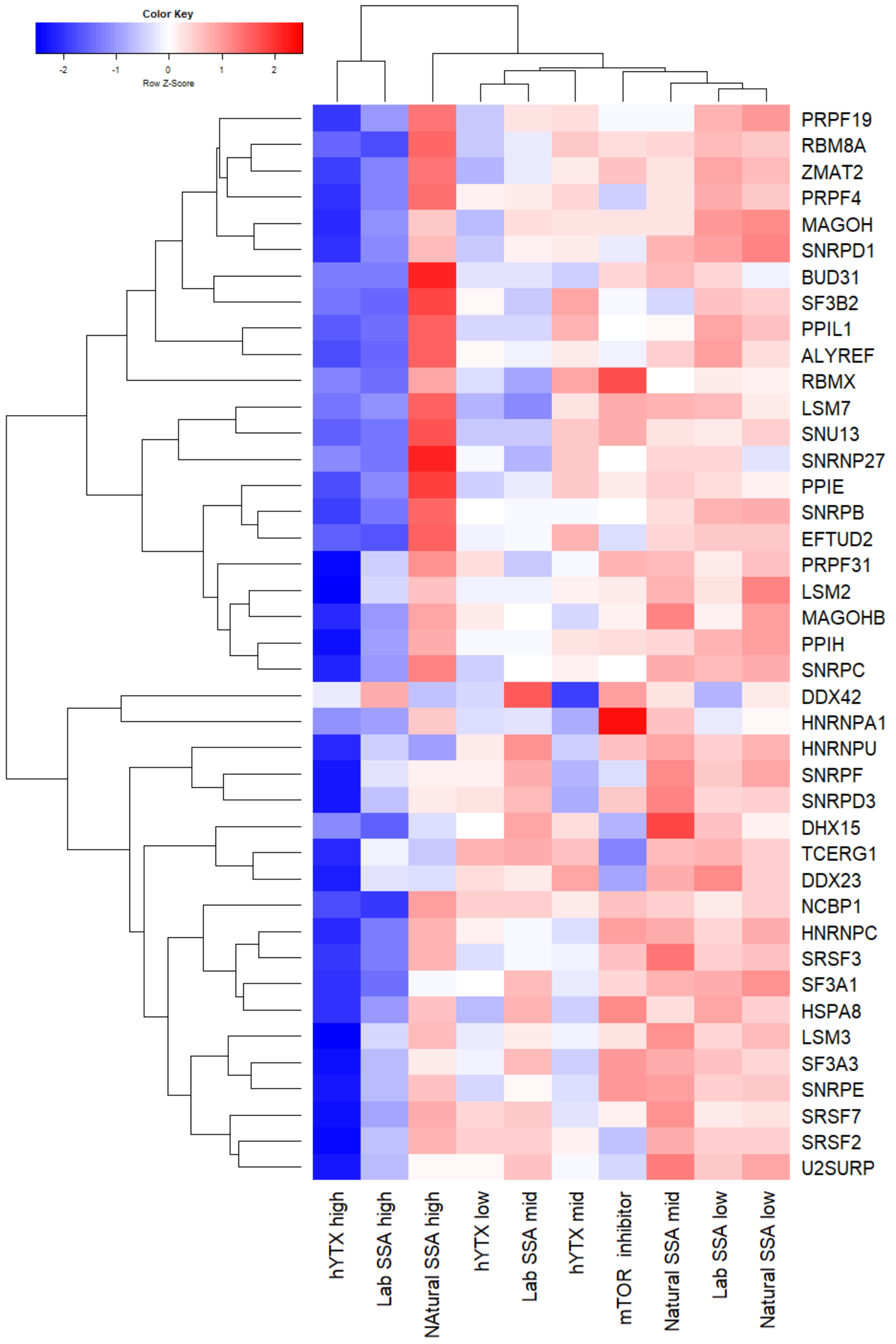

Figure S3: Heatmap of all genes with a significant dose response (FDR<0.01) effect in the spliceosome for all treatments and all dose levels (natural sea spray aerosol: natural SSA, laboratory sea spray aerosol: lab SSA, homoyessotoxin: hYTX, mTOR inhibitor).

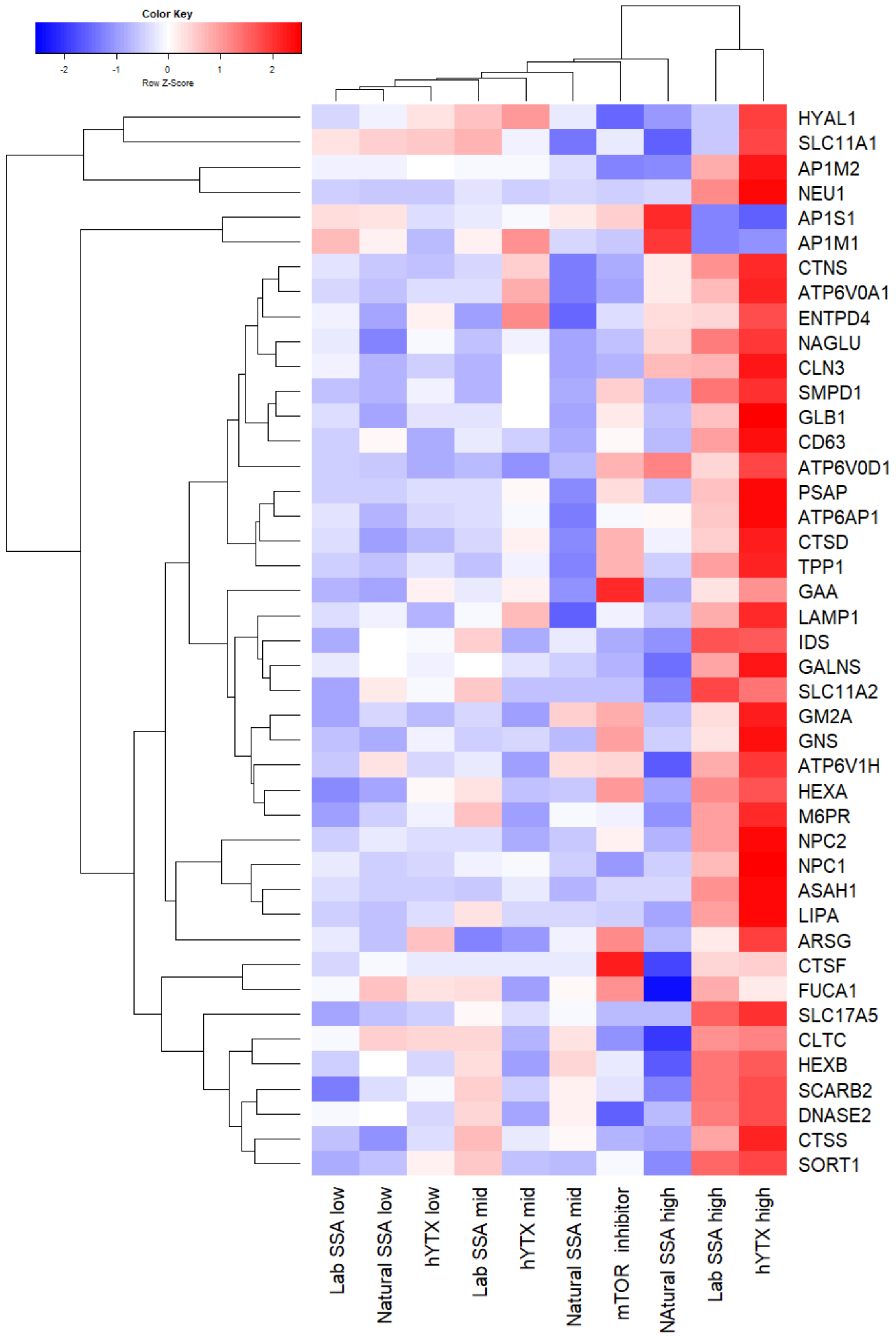

Figure S4: Heatmap of all genes with a significant dose response (FDR<0.01) effect in the lysosome for all treatments and all dose levels (natural sea spray aerosol: natural SSA, laboratory sea spray aerosol: lab SSA, homoyessotoxin: hYTX, mTOR inhibitor).

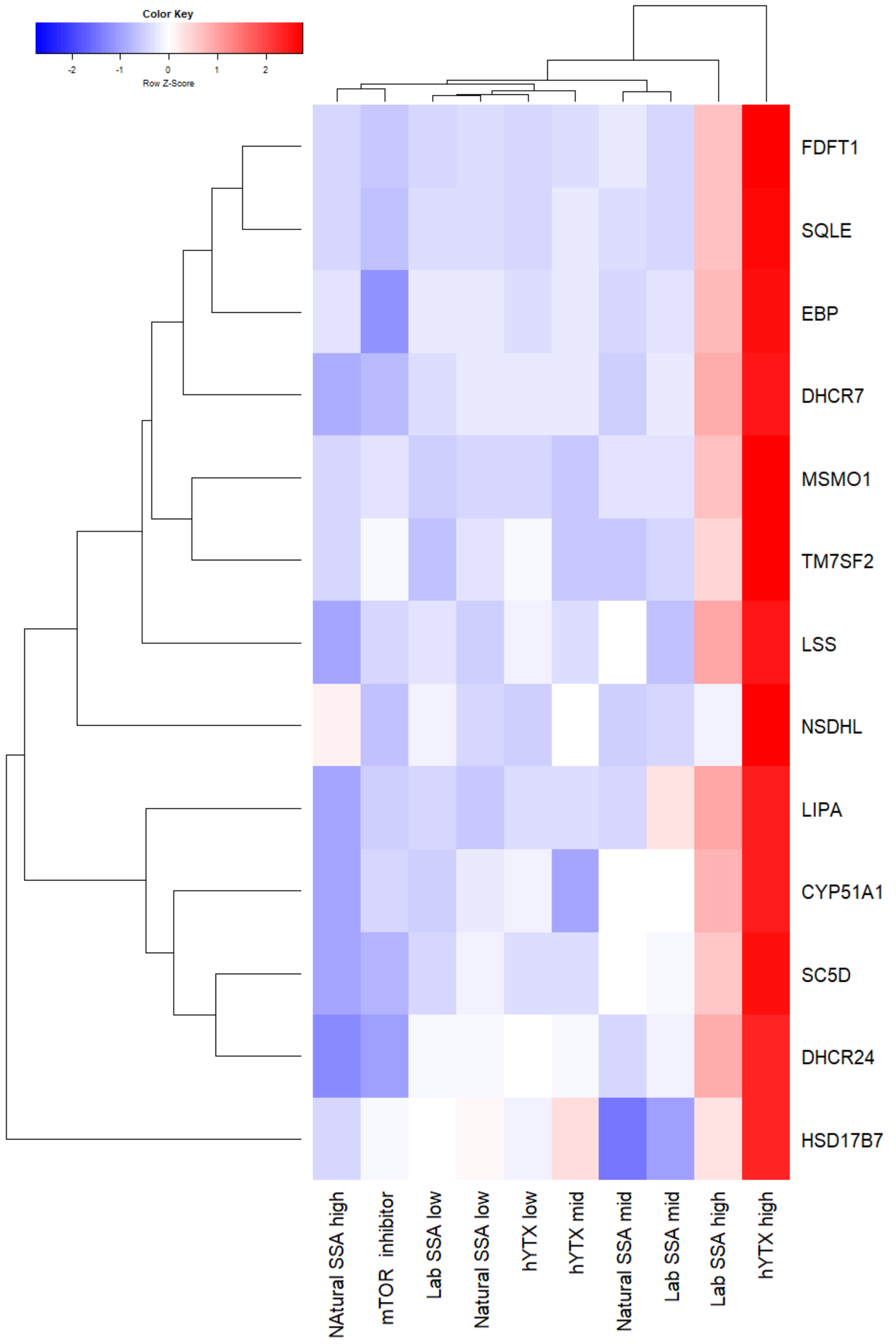

Figure S5: Heatmap of all genes with a significant dose response (FDR < 0.01) effect in the steroid biosynthesis for all treatments and all dose levels (natural sea spray aerosol: natural SSA, laboratory sea spray aerosol: lab SSA, homoyessotoxin: hYTX, mTOR inhibitor).

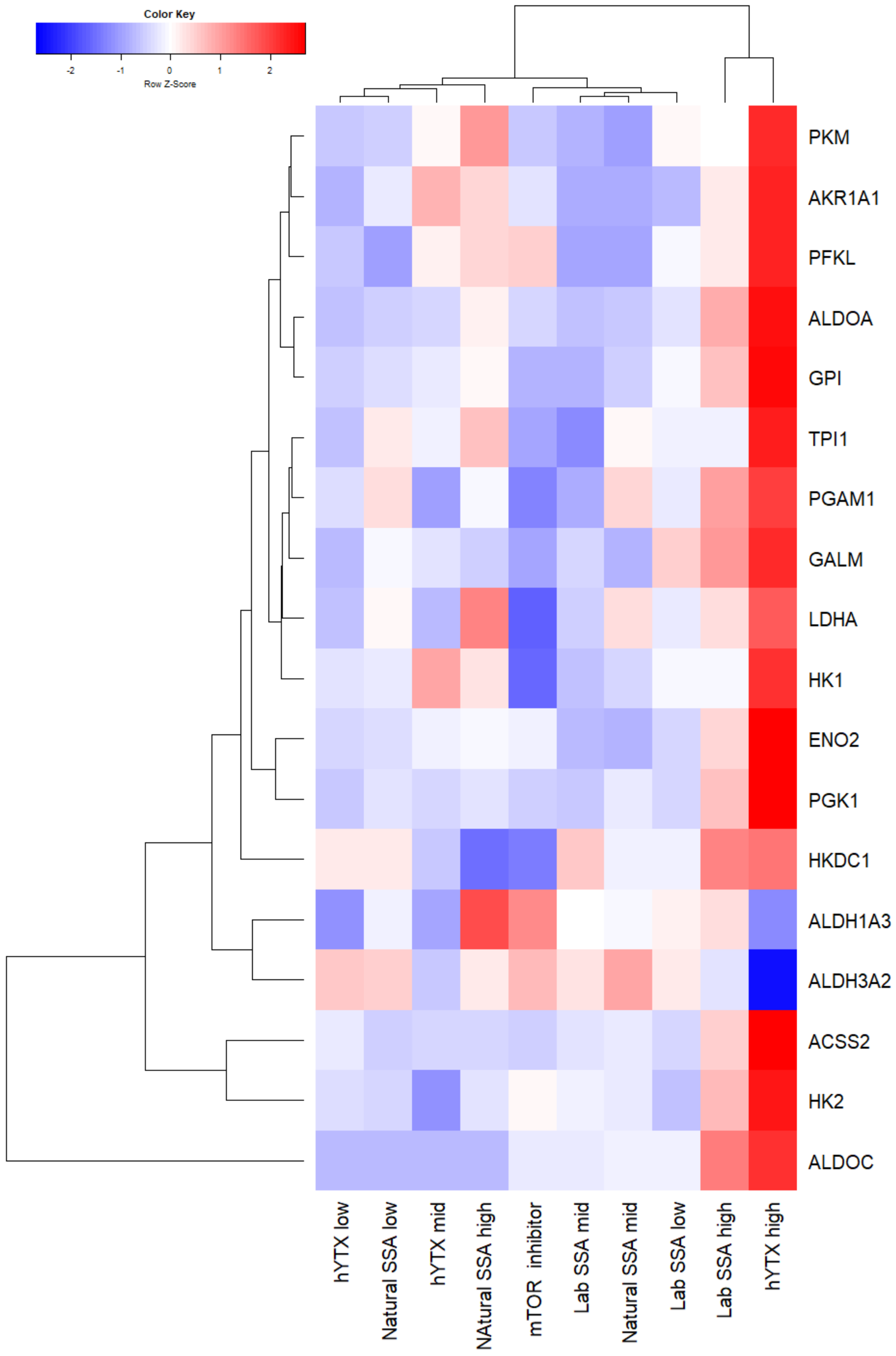

Figure S6: Heatmap of all genes with a significant dose response (FDR < 0.01) effect in the glycogenesis for all treatments and all dose levels (natural sea spray aerosol: natural SSA, laboratory sea spray aerosol: lab SSA, homoyessotoxin: hYTX, mTOR inhibitor).

References:

1 Orellana, G. *et al.* Validation of a confirmatory method for lipophilic marine toxins in shellfish using UHPLC-HR-Orbitrap MS. *Anal Bioanal Chem* **406**, 5303-5312, doi:10.1007/s00216-014-7958-6 (2014).

2 Koike, K. *et al.* Protoceratium reticulatum in northern Japan: environmental factors associated with seasonal occurrence and related contamination of yessotoxin in scallops. *J Plankton Res* **28**, 103-112, doi:10.1093/plankt/fbi103 (2006).

3 Akselman, R. *et al.* Protoceratium reticulatum (Dinophyceae) in the austral Southwestern Atlantic and the first report on YTX-production in shelf waters of Argentina. *Harmful Algae* **45**, 40-52, doi:10.1016/j.hal.2015.03.001 (2015).

4 Paz, B., Riobo, P., Ramilo, I. & Franco, J. M. Yessotoxins profile in strains of Protoceratium reticulatum from Spain and USA. *Toxicon* **50**, 1-17, doi:10.1016/j.toxicon.2007.02.005 (2007).

5 Riccardi, M. *et al.* Gonyaulax spinifera from the Adriatic sea: Toxin production and phylogenetic analysis. *Harmful Algae* **8**, 279-290, doi:10.1016/j.hal.2008.06.008 (2009).

6 Henke, K. G., Sharratt, M., Pegelow, D. & Dempsey, J. A. Regulation of end-expiratory lung volume during exercise. *J Appl Physiol (1985)* **64**, 135-146, doi:10.1152/jappl.1988.64.1.135 (1988).

7 Daigle, C. C. *et al.* Ultrafine particle deposition in humans during rest and exercise. *Inhal Toxicol* **15**, 539-552, doi:10.1080/08958370304468 (2003).

8 Frohlich, E., Mercuri, A., Wu, S. Q. & Salar-Behzadi, S. Measurements of Deposition, Lung Surface Area and Lung Fluid for Simulation of Inhaled Compounds. *Front Pharmacol* **7**, doi:ARTN 18110.3389/fphar.2016.00181 (2016).
